# Hemoglobinopathies in Iran: An Updated Review

**Published:** 2020-04-01

**Authors:** Abolfazl Nasiri, Zohreh Rahimi, Asad Vaisi-Raygani

**Affiliations:** 1 Students Research Committee, Kermanshah University of Medical Sciences, Kermanshah, Iran; 2 Department of Clinical Biochemistry, Kermanshah University of Medical Sciences, Kermanshah, Iran; 3 Medical Biology Research Center, Health Technology Institute, Kermanshah University of Medical Sciences, Kermanshah, Iran; 4 Fertility and Infertility Research Center, Health Technology Institute, Kermanshah University of Medical Sciences, Kermanshah, Iran

**Keywords:** Hemoglobinopathies, Thalassemia, Hemoglobin S, Hemoglobin D, Mutation

## Abstract

Hemoglobinopathies are the most common single gene disorders (monogenic disorders) in the world population. Due to specific position of Iran and the presence of multi-ethnic groups in the country, there are many varieties in the molecular genetics and clinical features of hemoglobinopathies in Iran. Hemoglobinopathies include structural variants, thalassemias, and hereditary persistence of fetal hemoglobin. In this review, we look at the common structural variants in various parts of the country along with their hematological and clinical characteristics. Also, we discuss about the burden of the thalassemias in the country, different types, complications, molecular defects and therapy.

## Introduction

 Hemoglobinopathies, inherited disorders of hemoglobin (Hb), are public health problem in the world. Hemoglobinopathies can be divided into structural variants, the thalassemias, and hereditary persistence of fetal hemoglobin (HPFH) ^   1 ^. Taken together, they are the most common single gene disorders (monogenic disorders) in the world population ^   1 ^.

 Various clinical manifestations of hemoglobin disorders can be attributed to the inﬂuence of environmental factors and various genetic modifiers. Heterogenous distribution of the disease and high variation in the phenotypic manifestation of a specific mutation are the main problems with the development of programs for the control of the hemoglobinopathies ^   2 ^. In Iran, the rate of hemoglobinopathies is high that could be attributed to the medium malaria endemicity that still exist in some provinces and high rate of consanguineous marriages in the country. So, the knowledge of genetic epidemiology and clinical features of hemoglobinopathies in the Iran will be valuable in prevention programs and better diagnosis and management of Hb disorders in the country ^   3 ^.

**Structural variants**

**The β-Chain variants**

Hb S as a beta chain variant results from glutamic acid → valine substitution at the 6^th^ codon of beta chain. This amino acid substitution in concentrated hemoglobin solutions and in the partial or fully deoxygenated conditions leads to polymerization and the occurrence of chronic hemolytic anemia and intermittent vaso-occlusive events (sickling disorders) ^4^^-^^6^ . These events result in tissue ischemia, which leads to acute and chronic pain as well as damage of different organs in the body ^   7 ^. The low prevalence of ischemic change in some patients may be partly explained by the higher Hb F percentage among them ^   8 ^. The sickle cell anemia (SCA) patients with high Hb F level, Southern Iran, India and Eastern Saudi Arabia have the benign clinical course  ^9^^, ^^10^ . 

The prevalence of sickle cell trait and SCA in southern Iran has been estimated to be 1.43 and 0.1%, respectively ^   11 ^, while in the center of Iran (Isfahan) the frequency has been reported to be 8.33% ^12^.

Blood transfusion is one of the most important treatments for sickle cell disease (SCD). Transfusion slows progressive hyperplasia in bone marrow and results in reduces the risk of heart failure and face and limb changes due to bone deformation  ^13^^-^^15^ . Some drugs such as hydroxyurea (HU) and 5-azacytidine by increasing formation of HbF are used in treatment the severity and the frequency of SCD episodes  ^16^^, ^^17^ .

The HbS has been found to be in linkage disequilibrium with five distinct common β-globin gene cluster haplotypes are known as African haplotypes (Benin, Bantu, Senegal, and Cameroon), and Arab-Indian haplotype ^   18 ^. In Iran, genetic studies for the first time in central and southwestern Iran indicated that the β^S^ disequilibrium gene was in linkage disequilibrium with the Arab-Indian haplotype in these regions  ^12^^, ^^19^ . The clinical presentation of SCA in southwestern Iran is associated with the elevation ratio of γ^G^ : γ^A^ chain and high level of Hb F in SCA patients that is related to Xmn I polymorphic site at 5´ to ε gene and is linked with Arab-Indian haplotype  ^20^^,^^21^ . However, in western Iran, the β^S^ gene is in linkage with the African haplotype of Benin ^   22 ^.

In 1951, another beta chain variant of hemoglobin, hemoglobin D (Hb D), was described. Variants of this Hb are Hb D-Bushman (β16 Gly→Arg), Hb D-Granada (β22 Glu→Val), Hb D-Ouled Rabah (β19 Asn→Lys), Hb D-Los Angeles or Hb D-Punjab (β121 Glu→Gln), Hb D-Iran (β22 Glu→Gln), Hb D-Ibadan (β87 Thr→Lys), ) and Hb D-Neath (β121 Glu→Ala). Only Hb D-Los Angeles and Hb D-Iran have been detected among Iranians. Hb D-Punjab was the most prevalent structural β-globin variant in Kurdish population from Western Iran and the second prevalent structural variant among Khuzestan province in Southern Iran  ^23^^, ^^24^ .

Hb D in homozygous state is accounted for 95% of Hb with normal Hb F and Hb A2 levels^   25 ^. Mild clinical presentation of Hb D-Punjab in homozygous and combined heterozygous state with β^0^-thalassemia mutation and also with α^0^-thalassemia mutations have been indicated ^   23 ^. In a report from South west of Iran, the combination of Hb D with β^0^ thalassemia presented with a benign nature ^   26 ^^.^

Molecular genetic studies in Western Iran demonstrated an association between Hb D-Punjab mutation with haplotype I [+ – – – – + +]. However, in southern Iran (Fars and Hormozgan provinces), β^D^ alleles were linked to four haplotypes, I, V [– + – – + + +], VII [+ – – – – – +], and IX [– + – + + + +] that among them the haplotype I (67.5%) was the most prevalent ^   27 ^. In Northern Iran, (Mazandaran province) three different haplotypes were linked to Hb D-Punjab. In most cases (91.4%) β^D^ alleles were associated with haplotype I [+– – – – + +] ^   28 ^.

**Common α-Chain variants**

Two variants of the α-globin gene including Hb Q-Iran and Hb Setif have frequently been found in heterozygous state among Iranians. Hb Q-Iran was introduced for the first time in 1970 by Lorkin et al. This Hb results from aspartic acid replacement by histidine at position α75 ^5^. Hb Q disorders including Hb Q-Iran [75 (EF4) Asp → His], Hb Q-India [64 (E13) Asp → His], and Hb Q-Thailand [74 (EF3) Asp → His]. These Hb variants slowly migrate with Hb S in electrophoresis at alkaline pH  ^29^^, ^^30^ .

Patients with Hb Q-Iran or Hb Q-India in heterozygous state do not show the thalassemia phenotype or any distinctive clinical manifestation ^   31 ^. Compound heterozygous state of Hb Q-Iran with a β^0^- thalassemia mutation and also in the presence of α^+^-thalassemia leads to a minor β-thalassemia (β-thal) picture with mild anemia and elevation of Hb F ^   32 ^. In carriers of Hb, Q-Iran hematological indices are normal and a level of 17–19% has been reported for this alpha chain variant of Hb ^29^. In studies from western Iran, this Hb variant was the second prevalent structural variant of Hb  ^22^^, ^^33^ .

Hb Setif [94 (G1) Asp → Tyr] is another α-chain Hb variant. This Hb has electrophoretic mobility similar to Hb S at alkaline pH  ^29^^, ^^30^ . In studies from Kurdish population of Western Iran this Hb variant was the third prevalent structural variant of Hb. The hematological indices of Hb Setif in heterozygote state are normal and the levels of 10.8 to 27.1% for this variant have been detected ^3^^, ^^33^^, ^^34^. A recent study reported a homozygous state of this Hb that produced anemia with persistent hypochromic microcytosis ^   35 ^.

**Thalassemias**

Thalassemias are divided into four types of α, β, γ and δ thalassemia. Around 1.7% of the world’s populations are carriers of α- or β-thalassemia. From each 10,000 live births, approximately 4.4% of them have thalassemia ^   36 ^. In Iran, there is around 2 million thalassemia carriers^   37 ^. Thalassemias are more prevalent in Northern and Southern regions of the country, where the carrier rate for α-thalassemia is around 35% and for β-thalassemia is about 10% ^   38 ^.

**β- Thalassemia**

β-thalassemia is an autosomal recessive inherited disorder due to decreased or the absence of β-globin chain production. There are 200 mutations linked with a β-thalassemia phenotype that affect the stages of β-globin gene expression and cause a reduction (β^+^) or complete absence (β^0^) of β-chain synthesis^39^^,^^40^ .

This hematological disorder has a high prevalence among Asian, Indian, Middle Eastern and Mediterranean populations^   41 ^. During prenatal diagnosis (PND) programs in Iran, more than 52 thalassemic mutations with different ethnic heterogeneity have been detected ^42^^,^^43^. 

In three Northern provinces of Gilan, Mazandaran and Golestan, the IVS-II-1 G→A was the most prevalent (56.1%) and the CD 30 G→C (8.1%) was the second prevalent β-thalassemic mutations ^73^. However, in more recent study in Mazandaran and Golestan provinces of Northern Iran, the IVSII-74 (G/T) with a frequency of 54.71% was the most prevalent mutation ^   45 ^. In Northeastern province of Khorasan, the CD 8/9 +G was the most prevalent mutation (62.5%), and the second prevalent mutations were IVS-II-1 G→A, 36/37 (-T), and CD 39 C→T, each had equal frequency of 12.5% ^43^. In more recent study in this province, the IVS-I-5 G→C (42.03%) was the most prevalent mutation and codon 8/9 +G had a frequency of 4.79% ^   46 ^. In Northwestern province of Tabriz, codon 36 / 37 (-T) was found to be the most prevalent mutation ^   44 ^.

In Southern provinces, the IVS II-I G→A, IVS I-5 G→C, C36–37 (-T), 25bp del (252–276), IVS I-110 G→A and C44 (-C) were the major common mutations responsible for β-thalassemia mutations in Southern Iran ^   47 ^. In Southeastern Iran, among Balouch population, the IVS I- 5 G→C with a frequency of 87.2% and CD 8/9 +G with a frequency of 4% constituted about 91% of β-thal mutations ^   48 ^. Also, in Southeastern province of Kerman, the IVS I-5 G→C was the highest prevalent β-thalassemia mutation (66.2%) ^   49 ^.

In western Iran provinces of Kermanshah, Kurdistan, Ilam (mostly Kurds), Hamadan (mostly Fars) and Lorestan (mostly Lors), β-thalassemia mutations were identified  ^50^^-^^52^ . In Kermanshah province, the most common mutation was the IVSII-1 G→A (32.97%) ^   51 ^. In the Kurdistan province, the most common mutation was found to be IVS-II-1 G→A (35%)^52^. In the Lorestan province, the CD 36/37 (-T) mutation with a frequency of 33.8%, and in two provinces of Hamadan and Ilam the IVSII-1 G→A with a frequency 29.4% were the most prevalent mutations ^   50 ^^.^

**Types of β-thalassemia**

According to clinical manifestations, the β-thalassemia is classified into three types of β- β- β-thalassemia minor (β-thal minor), β-thalassemia intermedia (β-TI) and β-thalassemia major (β-TM) ^   53 ^. 

β-thal minor is due to a single mutation in β-gene, which leads to decrease biosynthesis of Hb A (α2β2)  ^54^^, ^^55^ . Due to the presence of excess and unmatched *α* chains, red blood cell (RBC) destruction increases that leads to decreased Hb level. The β-thal minor patients are asymptomatic since one β-globin gene still is normal and the clinical condition in these patients is mild-to-moderate microcytic anemia ^56^. The β-thal minor patients usually experience bone pain complaint, muscle weakness, myalgia and fatigue ^   57 ^. Abnormal low plasma carnitine concentrations which lead to deficient ATP production, fatigue and bone pain complaint has been reported in these patients and carnitine and folic acid supplementation lead to a decrease in muscle weakness and bone pain complaint ^   58 ^. 

β-thalassemia intermedia.Genetic heterogeneity of β-TI is associated with wide clinical spectrum manifestation from mild to severe hemolytic anemia. Based on the clinical symptoms of β-TI, it can be divided into two subgroups: some patients are mildly aﬀected with mild clinical problems until adult life. In this subgroup, Hb levels maintain between 7 and 11 g/dL and are usually rarely require blood transfusion ^   59 ^. The second subgroup consisted of patients that have severe anemia which generally present at ages 2–6 years old. These patients frequently develop clinical symptoms such as growth retardation and skeletal deformities  ^60^^-^^62^ . These patients are usually diagnosed after the age of 2 years with Hb levels of 7 g/dL or free of infection and with adequate folic acid. In some carriers of this disease, normal or borderline HbA2 or isolated increased HbF is observed (up to 10%)  ^60^^-^^62^ . Diﬀerential diagnosis between β-TI and β-TM is essential ^63^ since the first choice of β-TM management is blood transfusion, while the first step for management of patients with β-TI is usually not transfusion. In these patients, the hydroxyurea (HU) therapy, blood transfusion, and radiation therapy are therapeutic options. There are several reports indicating that erythropoietin, HU (an Hb F augmenting agent), and Minihepcidin Peptide or similar drugs (ACE-536, ACE-011), which promote RBC differentiation or maturation in the bone marrow improve anemia  ^64^^-^^67^ . The dosage of HU which can be eﬀective and safe in β-TI for enhancement of gamma globin chain synthesis is 8–15 mg/kg/d. In patients with β-TI, the HU therapy in combination with magnesium or L-carnitine can be eﬀective in improving hematologic parameters and cardiac status  ^68^^,^^69^ . No significant association between HU response and single-nucleotide polymorphism in β-TI patients has been detected ^   70 ^.

β-TM is usually diagnosed in the first 2 years of life with severe anemia, poor growth and skeletal abnormalities. Untreated β-TM usually leads to heart failure and consequently death ^   44 ^. The first step for management of patients with β-TM is blood transfusion. Blood transfusion leads to iron overload and its complications such as cardiac and liver dysfunction, immune impairment, and endocrine deficiencies ^   59 ^. Iron chelators such as deferoxamine , deferiprone, and deferasirox can reduce the excess iron in the body and prevent serious complications in patients with β‐TM ^   71 ^. Deferoxamine is the standard treatment for iron overload. Because of the complications of these drugs, new studies are focused on using natural iron chelating agents  ^72^^-^^74^ . So, a recent study has suggested silymarin (a flavonoid extract from the Silybum marianum) as an iron chelator could be useful ^   75 ^. Some micro RNAs (miRNAs) can regulate the maturation and the proliferation of erythroid cells, and also the expression of fetal γ-globin genes. Using miRNA for treatment of β-TM indicated a significant increase in γ-globin gene expression in the responder group ^   76 ^. However, due to high cost of health care for β-TM treatment and the lack of suitable treatment, the PND is the best way to control the prevalence of the disease. Termination of pregnancy has been allowed in Iran since 2000 in a fetus with genetic disorder ^   47 ^. Evaluating the outcome of the PND has indicated that it is an integrated primary health care approach with best infra-structure for implementing successful strategies that significantly reduced the rate of β-thalassemia ^77^. Studies are now looking for novel methods with high sensitivity and specificity for detection of a paternally inherited mutation in a fetus ^   78 ^. It has been suggested that the real-time PCR high-resolution melt could be a sensitive and specific method for distinguish the paternally inherited mutation in a fetus at risk with β-TM ^   79 ^. 

**α-Thalassemia**

**Figure1:**
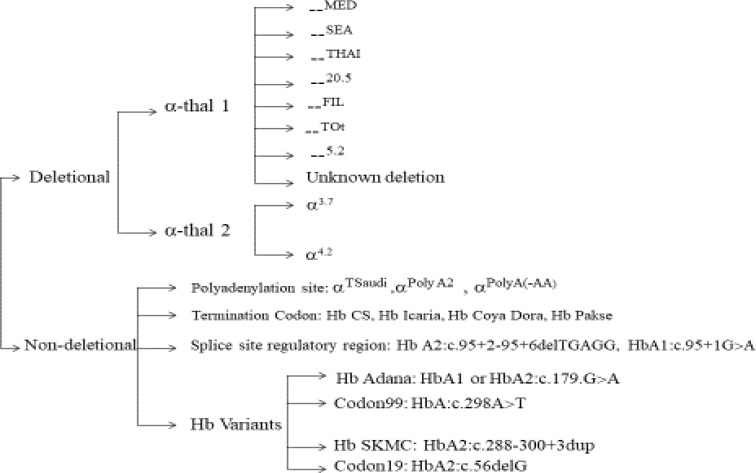
Frequently deletional and non-deletional mutations involved α-thalassemia are presented in tree diagram ^   101 ^.

α-Thalassemia is a hereditary autosomal recessive disorder resulting from deletions or mutations within the α-globin gene cluster including of two alpha 1 (α1) and alpha 2 (α2) globin genes that are located on chromosome 16p13. More than 750 different variants in α-globin genes have been identified, leading to α-thalassemia worldwide ^80^. It is estimated that more than 5.0% of the world’s population are carriers of -thalassemia^   81 ^. The α-thalassemia is commonly found in sub-Saharan Africa, Mediterranean region, Middle East, Indian Subcontinent, East, and Southeast Asia and immigrants to these areas  ^82^^-^^85^ . Middle East is so-called thalassemia belt. Iran is located in the Middle East between Iraq and Pakistan, and the incidence of α-thalassemia in Iran is high ^50^^, ^^86^ . Although the frequency of α-thalassemia carriers in Iran is not well detected, one report from Northern Iran has estimated its frequency around 15.0% ^   87 ^. In Iran, more than 19 different α-globin gene mutations have been identified, representing the heterogeneity of the population ^88^^,^^89^. Common and rare mutations of α-thalassemia can be classified into deletional, and non-deletional. The most common deletional and non-deletional mutations are shown in Figure 1. Over 70 non-deletional forms of α-thalassemia have been detected that co- inherited with deletional mutations (90) or with other genetic modifiers, leading to diverse genotypic and/or phenotypic expressions ^   91 ^. The spectrum of α-thalassemia mutations in different regions of Iran showed that the α^-3.7^ (rightward deletion), – –^MED^ (Mediterranean deletion) and α^-4.2^ (leftward deletion) are the major common mutations among Iranian patients  ^88^^, ^^92^ . Kerman province has the highest frequency of α ^3.7^ deletion among Iranian population with a frequency of around 83% ^   93 ^. However, in Gilan and Mazandaran (two Northern provinces), the frequency of α ^3.7^ deletion are lower than others, 42.5 and 44.9%, respectively ^94^. This high prevalence of the α ^3.7^ deletion could be due to the high rate of consanguine marriages among Iranians ^   94 ^. The second most common mutation in other parts of Iran is different as in the Mazandaran province (Northern province) the α^polyA2^ is the second prevalent mutation ^   95 ^. However, in Khuzestan province (Southwest Iran) – –^MED ^^96^, and in Hormozgan and Kermanshah provinces (Southern and Western Iran, respectively) the α^5nt ^^97^^, ^^98^  is the second most common mutations. The presence of α-thalassemia 1 and α-thalassemia 2 in trans position (- -/-α) is the classic form of HbH disease known as deletional HbH disease. The α^-3.7^ (single deletion) and - -^20.5^ kb and - -^MED^ (double deletions) are reported as the most deletions among Iranian HbH patients, while the α^-3.7^, α^-4.2 ^, - -SEA, - -^MED^, - -^THAI^, - - ^20.5^, - - ^Tot^, - - ^FIL^ and --^5.2^ are the most observed mutations of HbH disease in different populations ^89^^, ^^90^^, ^^99^ . The most common genotype among Iranians is α^3.7^/- -^MED ^^   100 ^. 

In α-thalassemia carriers, the levels of mean corpuscular volume and mean corpuscular hemoglobin decreased, and the Hb A2 level was normal or slightly decreased along with normal level of Hb F ^   102 ^. Clinical severity of the of α-thalassemia depends on the type of mutation (deletional or non-deletional) and the copy number of affected α- gene ^   103 ^. 

By timely screening, Hb Bart’s hydrops fetalis (four defective α-globin genes) or Hb H disease (three defective α-globin genes) can be diagnosed during prenatal. Blood transfusion is by far the most important treatment for patients with thalassemia ^4^^–^^10^, but the frequency of blood transfusion varies depending on the type of α-thalassemia. Patients with non-deletion type of Hb H disease have more symptoms at younger age and need more transfusions than patients with deletional Hb H disease  ^100^^, ^^104^^, ^^105^ . In spite of the vital role of transfusion, it is associated with iron overload and adverse reactions in the recipients ^   106 ^. Adverse transfusion reactions can be divided into acute and delayed reactions, the acute reactions (more common) occurring within the first 24 hours of transfusion, and delayed reactions occurring after the first 24 hours. Hemovigilance is a set of supervision activities that is used to monitor and assess the safety of blood transfusions from donors to recipients, and the improvement of process and training of staff ^107^^,^^108^. This system was introduced in Iran in 2009, which has been used in a study in Shiraz ^   106 ^.

## CONCLUSION

 Due to specific location of Iran and the presence of various ethnic groups in the country, there are many varieties in the molecular genetics and clinical features of hemoglobinopathies in the country. Hemoglobinopathies included structural variants, thalassemias, and HPFH. Many structural variants have been identified in Iran, but among these abnormal variants, β-globin chain variants of Hb S and Hb D and α-globin chain variants of Hb Q-Iran and Hb Setif are more common. Thalassemia is one of the major genetically inherited hematological diseases. A wide spectrum of β-thalassemia alleles has been detected among Iranians with IVSII-1 G→A as the most prevalent β-thalassemia mutation. Among Iranians, more than 19 different α-globin gene mutations have been detected, which represent the heterogeneity of the population. The α-^3.7Kb ^was found to be the major common deletional mutation among Iranians. The first step for management of patients with severe form of thalassemia is blood transfusion; however, it leads to an iron overload and its complications. So, new therapies have recently been proposed for the disease.
